# Supplementary material for: Lecturers’ readiness for EMI in Malaysia higher education
Source: PLoS One. 2023 Jul 26;18(7):e0284491. doi: 10.1371/journal.pone.0284491 (PMC10370753; doi:10.1371/journal.pone.0284491)

Raw data


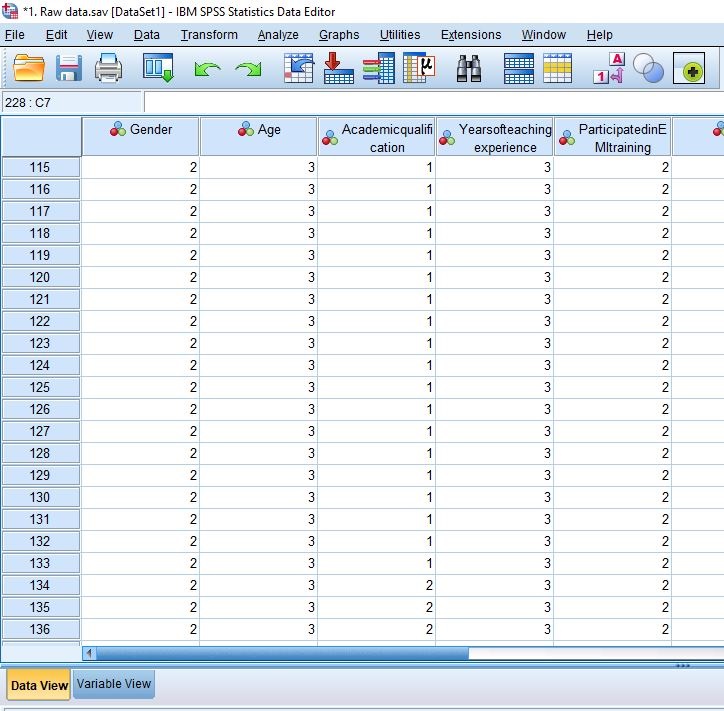


Table 7: Dimension 1 – knowledge and understanding by gender


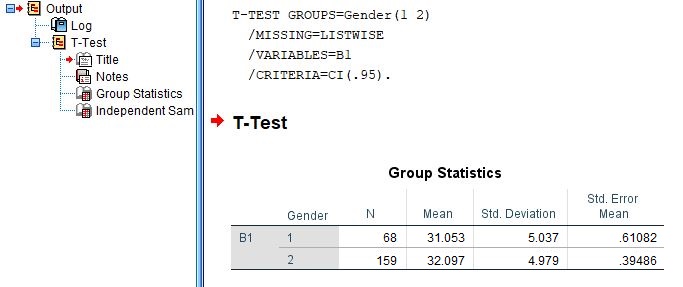


Table 7: Dimension 2 – skills and abilities by gender


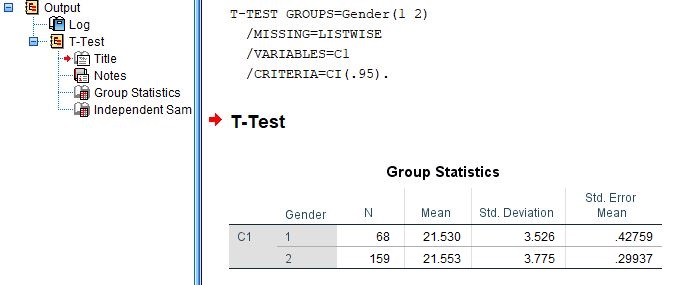


Table 7: Dimension 3 – attitudes by gender


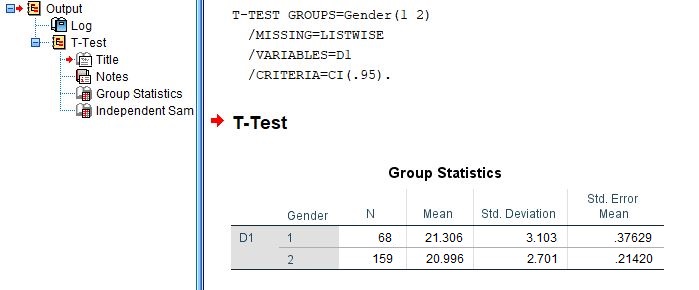


Table 8: readiness by age


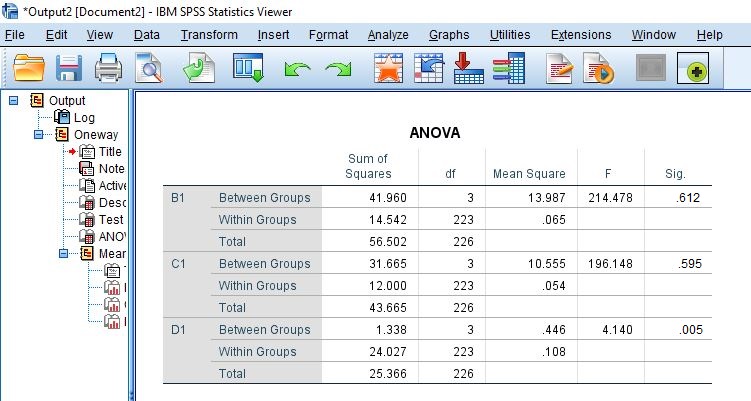


Table 9: readiness by academic qualification


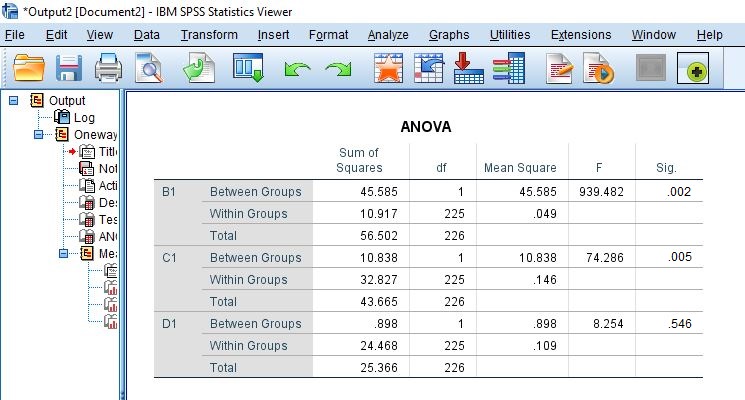


Table 10: readiness by years of teaching experience


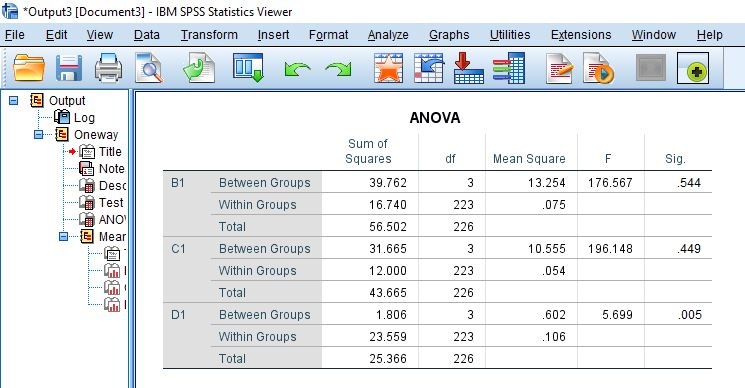


Table 11: readiness by experience in teaching through EMI


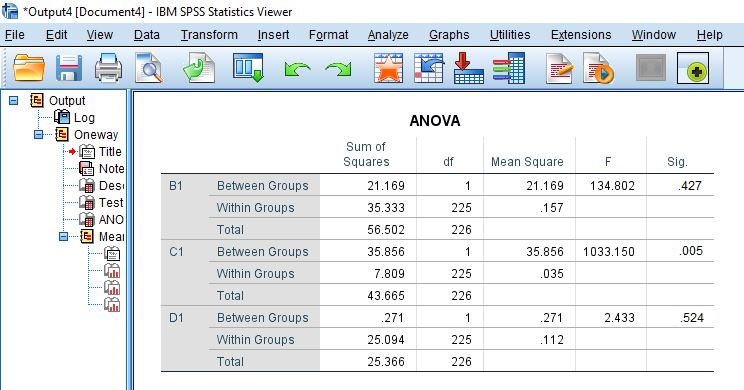


Table 12: readiness by EMI training


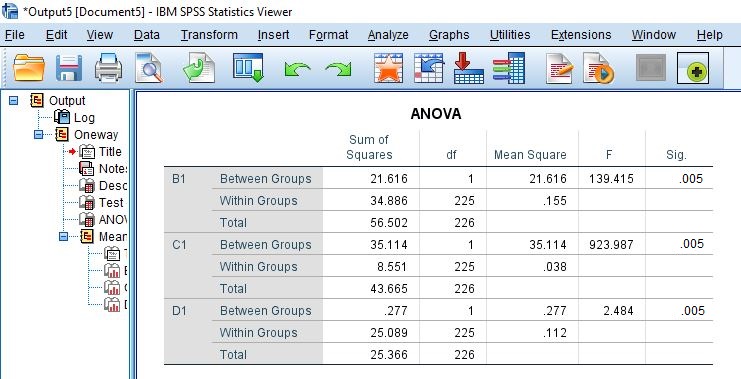

Supplement: S1 Raw data — (DOCX) [file pone.0284491.s001.docx]
