# Supplementary material for: Lecturers’ readiness for EMI in Malaysia higher education
Source: PLoS One. 2023 Jul 26;18(7):e0284491. doi: 10.1371/journal.pone.0284491 (PMC10370753; doi:10.1371/journal.pone.0284491)
Supplement: S1 File — (DOCX) [file pone.0284491.s002.docx]

**Permission to Inviting Lecturers to Participate in Survey Research**

Dear respected Head of department _________________________,

**Subject: Seeking permission to invite lecturers from your department to participate in survey research**

I would like to ask your permission to allow me to invite all the lecturers in your department to take part in a study about EMI in Malaysian higher education. With this research project, we hope to learn more about the lecturers’ readiness, preparation, and support for EMI faculty.

I, Dr Janice Lo from the Universiti Malaya will carry out this research.

Participation in this survey research is completely voluntary. The lecturers can choose whether to take part or not. If any decline to participate in this survey research, no one in the campus will be told. All information will be handled with the utmost confidentiality and anonymity. The study will never associate a survey respondents’ name or ID, institutions, and departments in any form of reporting.

**Please note**: The four (4) sections of this survey study project are as follows. They are:

Section A: Demographics (6 questions)

Section B: Lecturers’ knowledge and understanding about EMI in higher education (8 questions)

Section C: Lecturers’ skills and abilities pertaining to EMI (8 questions)

Section D: Lecturers’ attitudes towards EMI in higher education (8 questions)

There are no known risks associated with taking part in this survey research. If you have any questions at any time, you may contact me at [janice@um.edu.my](mailto:janice@um.edu.my).

If you agree to grant the permission for inviting all the lecturers to participate in this survey research, please sign the attached permission letter.

I would be grateful if you could grant the permission to invite all your lecturers in your department to participate in this survey research.

Thank you for your time and consideration.

Yours sincerely,

Dr. Janice Lo

Universiti Malaya

_________________________________________________________________________

I have read the above information and offer permission to conduct this survey research.

________________________________ (Signature)

________________________________ (Date)
