# Supplementary material for: Lecturers’ readiness for EMI in Malaysia higher education
Source: PLoS One. 2023 Jul 26;18(7):e0284491. doi: 10.1371/journal.pone.0284491 (PMC10370753; doi:10.1371/journal.pone.0284491)
Supplement: S2 File — (DOCX) [file pone.0284491.s003.docx]

**Consent for Participation in Survey Research**

Dear esteemed lecturers,

You are invited to take part in a study about EMI in Malaysia higher education. The purpose of this research study is to shed light on the lecturers’ readiness and support requirements for EMI faculty and the lecturers’ preparation.

This study is conducted by Dr. Janice Lo (janice@um.edu.my) from University of Malaya.

Participation in this survey research is voluntary. You can choose whether to take part or not. If you decline to participate in this survey research, no one in the campus will be told.

**Please note**: The four (4) sections of this survey study project are as follows. They are:

Section A: Demographics (6 questions)

Section B: Lecturers’ knowledge and understanding about EMI in higher education (8 questions)

Section C: Lecturers’ skills and abilities pertaining to EMI (8 questions)

Section D: Lecturers’ attitudes towards EMI in higher education (8 questions)

There are no known risks associated with taking part in this survey research. If you have any questions at any time during the study, you may contact me at [janice@um.edu.my](mailto:janice@um.edu.my).

If you agree to participate in this survey research, please sign the attached informed consent form

Thank you for your help.

Sincerely,

Dr. Janice Lo

Universiti Malaya

_________________________________________________________________________

I have read the above information and consent to voluntarily participate in this survey research.

________________________________ (Name)

________________________________ (Signature)

________________________________ (Date)

**Lecturers’ Readiness for EMI in Malaysia Higher Education**

**Section A: Demographics**

**Please tick the appropriate column for each question.**

**1. Gender**

| Male |  |
| --- | --- |
| Female |  |

**2. Age**

| 25-29 |  |
| --- | --- |
| 30-34 |  |
| 35-39 |  |
| >40 |  |

**3. Academic Qualification**

| Master’s degree |  |
| --- | --- |
| Doctoral Degree |  |

**4. Years of Teaching Experience**

| <5 |  |
| --- | --- |
| 6-10 |  |
| 11-15 |  |
| >15 |  |

**5. Years of Experience in Teaching through EMI**

| 1 year or less |  |
| --- | --- |
| More than 1 year |  |

**6. Participated in EMI Training?**

| Yes |  |
| --- | --- |
| No |  |

**Section B: Lecturers’ knowledge and understanding about EMI in higher education**

**Please indicate the extent to which you agree or disagree with the following statements using the scale below.**

| 1 | 2 | 3 | 4 |
| --- | --- | --- | --- |
| Strongly Disagree | Disagree | Agree | Strongly Agree |

| No. | Items | 1 | 2 | 3 | 4 |
| --- | --- | --- | --- | --- | --- |
| 1 | I understand the meaning of EMI in higher education. |  |  |  |  |
| 2 | I understand the purpose of EMI in higher education. |  |  |  |  |
| 3 | I understand the process involved in teaching academic subjects through EMI. |  |  |  |  |
| 4 | I understand the university curriculums and policies on EMI in higher education. |  |  |  |  |
| 5 | I have the knowledge to create a conducive learning environment in an EMI classroom. |  |  |  |  |
| 6 | I have knowledge of how to teach students with diverse needs and experiences in the EMI classroom. |  |  |  |  |
| 7 | I have the knowledge to sustain the learning in the EMI classroom. |  |  |  |  |
| 8 | I have knowledge of assessing students with diverse needs and experiences in the EMI classroom. |  |  |  |  |

**Section C: Lecturers’ skills and abilities pertaining to EMI**

**Please indicate the extent to which you agree or disagree with the following statements using the scale below.**

| 1 | 2 | 3 | 4 |
| --- | --- | --- | --- |
| Strongly Disagree | Disagree | Agree | Strongly Agree |

| No. | Items | 1 | 2 | 3 | 4 |
| --- | --- | --- | --- | --- | --- |
| 1 | I am able to teach students with diverse needs and experiences through EMI. |  |  |  |  |
| 2 | I am able to explain the course material well to students with diverse needs and experiences through EMI. |  |  |  |  |
| 3 | I am able to discuss with students the subject issues at length with relative ease and accuracy through EMI. |  |  |  |  |
| 4 | I am able to discuss with students regarding the English-language-related challenges through EMI. |  |  |  |  |
| No. | Items | 1 | 2 | 3 | 4 |
| 5 | I am able to discuss with students the strategies needed to follow the lecture delivered through EMI. |  |  |  |  |
| 6 | It is hard to control students with diverse needs and experience in EMI classroom. |  |  |  |  |
| 7 | I need support from English language lecturers if I have to teach students with diverse needs and experiences through EMI. |  |  |  |  |
| 8 | I need extra effort to teach students with diverse needs and experience through EMI. |  |  |  |  |

**Section D: Lecturers’ attitudes towards EMI in higher education**

**Please indicate the extent to which you agree or disagree with the following statements using the scale below.**

| 1 | 2 | 3 | 4 |
| --- | --- | --- | --- |
| Strongly Disagree | Disagree | Agree | Strongly Agree |

| No. | Items | 1 | 2 | 3 | 4 |
| --- | --- | --- | --- | --- | --- |
| 1 | I care for the progressive learning of students with diverse needs and experiences in the EMI classroom. |  |  |  |  |
| 2 | I care for the achievement of students with diverse needs and experiences in the EMI classroom. |  |  |  |  |
| 3 | I care for the skill advancement of students with diverse needs and experiences in the EMI classroom. |  |  |  |  |
| 4 | I believe students with diverse needs and experiences in the EMI classroom can achieve their best with support. |  |  |  |  |
| 5 | I believe students with diverse needs and experiences in the EMI classroom can be equipped with both English and subject knowledge. |  |  |  |  |
| 6 | I believe students with diverse needs and experiences in the EMI classroom can adopt learning strategies to compensate for comprehension problems with support. |  |  |  |  |
| 7 | Students with diverse needs and experiences will interrupt the teaching and learning process through EMI. |  |  |  |  |
| 8 | I do not feel confident and comfortable teaching academic subjects through EMI. |  |  |  |  |

- The End -
