# Supplementary material for: Lecturers’ readiness for EMI in Malaysia higher education
Source: PLoS One. 2023 Jul 26;18(7):e0284491. doi: 10.1371/journal.pone.0284491 (PMC10370753; doi:10.1371/journal.pone.0284491)
Supplement: Attachment — Submitted filename: Response to reviewers.docx [file pone.0284491.s004.docx]

**Dear Editor of PLOS ONE:**

We would like to express our gratitude for the opportunity to submit a revised manuscript in response to the reviewers’ insightful comments and recommendations. We have considered all of the comments by addressing each comment and suggestion one at a time. Also, we have ensured that a reference to Table 7 is included in our text (manuscript and revised manuscript with track changes).

Last but not least, we have proofread the manuscript and submitted the needed files in the revised format. We wish to thank the PLOS ONE editor and the reviewers for their efforts to make this manuscript better. We greatly appreciate it.

**Editors’ comments to the authors**

**Comment 1**: A rebuttal letter that responds to each point raised by the academic editor and reviewer(s). You should upload this letter as a separate file labelled ‘Response to Reviewers’.

**Response**: Editor, thank you for your comment and suggestion. The authors have attached a rebuttal letter, as per your suggestion.

**Comment 2**: A marked-up copy of your manuscript that highlights changes made to the original version. You should upload this as a separate file labelled ‘Revised Manuscript with Track Changes’.

**Response**: Thank you very much, editor. As per your suggestion, the document with tracked changes has been prepared, labelled, and attached.

**Comment 3**: An unmarked version of your revised paper without tracked changes. You should upload this as a separate file labelled ‘Manuscript’.

**Response**: Thank you, kindly, editor. The document without tracked changes has been uploaded as a separate file as per your suggestion.

**Journal requirements**

**Comment:** If you would like to make changes to your financial disclosure, please include your updated statement in your cover letter. Guidelines for resubmitting your figure files are available below the reviewer comments at the end of this letter.

**Response**: Thank you for your suggestion and comment, editor. We agreed with your observation. The financial disclosure would remain unchanged because the author(s) received no specific funding for this work. We have made certain that all relevant data are within the manuscript and its supporting information.

**Reviewers comment to the authors**

**Comment Reviewer#1:** There is moderate level of role of lecturers’ readiness for EMI regarding knowledge, understanding, skills, abilities, and attitudes. Since English is a second language in Malaysia, Malaysian Ministry of Education has to make policy decision for lecturers’ readiness for EMI by undergoing training or their selection criterion should have EMI trained persons to impart better quality of education to enhance cultural communicative competence in many increasingly diversified student bodies in EMI classroom. Difference existed in lecturers’ readiness based on gender, age, academic qualification teaching course, teaching involvement, and EMI training. Experience lecturers manifested more negative attitude towards EMI. Elder lecturers had high degree of negative attitude towards higher education EMI. The study should be carried out throughout Malaysia including all lecturer’s readiness for EMI and for better conclusion of having trained teachers with positive attitude towards readiness for EMI knowledge and for training of EMI.

**Response**: Thank you so much. We really appreciate your feedback and ideas, reviewer. The authors agreed and gladly accept your suggestion. Hence, we revised the conclusion section in the revised manuscript. The changes made in the conclusion section in red font colour as a response to this comment are:

*“The study has significant implications for stakeholders in Malaysian universities and other emerging EMI contexts because it reveals how cultural communicative competence may be effectively integrated as a vital learning and teaching tool within specific EMI disciplinary curricula. The data presented here emphasises the dangers of using ‘one-size-fits-all’ approaches to language policy in diverse multilingual EMI contexts. Since English is a second language in Malaysia, the Malaysian Ministry of Education ought to make policy decisions regarding lecturer readiness for EMI through training or include EMI trained individuals in their selection criteria to impart better quality education and enhance cultural communicative competence in EMI classrooms.”*

*“Likewise, it would be beneficial to explore lecturers’ readiness for EMI throughout Malaysia to get a better conclusion of having trained lecturers with a positive attitude toward EMI knowledge and training.”*

**Comment Reviewer#2:** The authors’ examined lecturer readiness to teach via implementation of the English Medium Instruction (EMI) based on self-reporting of lecturer participants. It is unclear what the latter part of the title, “Fantasy or realities?” reference for the purpose of the study. Based on participant responses, the overarching conclusion indicated that lecturers with more knowledge, understanding, and experience in EMI were more accepting of the practice. Thus, following the conclusion, it would be beneficial to know what recommendations the authors have in advancing and facilitating more positive acceptance and readiness of lecturers for EMI at all levels, i.e., what professional development opportunities or programs would be offered? The study was limited to two private higher institutions and did include representative sample from public institutions of higher education in Malaysia – the conclusion only noted that “current study limitation was the employment of self-reported questionnaire, which potentially reduced social desirability response bias.” Please see additional comments below.

**Response**: Thank you very much, reviewer. We value your advice and are grateful that you pointed out ways we could improve this manuscript. First, we omitted the latter part of the title “Fantasy or realities?” because the phrase does not adequately reflect the aim of this study and detracts from the seriousness of this investigation.

Second, we revised the conclusion section to incorporate our recommendations for encouraging and facilitating lecturers' readiness for EMI as suggested as well as more positive acceptance:

*“Based on the study findings, two recommendations can be made, which are intended to serve as a foundation for any EMI lecturer training and continuing professional development at Malaysian higher education institutions. First, the EMI lecturer training programs in applied sciences, social sciences, and humanities should be more context-bound and needs-based, taking into account the perspectives of EMI lecturers as insiders. Second, EMI training programs should have two key components: pedagogical and linguistic training, with the former focusing on teaching techniques, strategies, and classroom management and the latter on language-related issues. To improve lecturers’ reflection on their own classroom delivery ad practices, pedagogical training should use a variety of strategies (e.g., peer observation, coaching, and mentoring between senior and younger EMI lecturers), while linguistic training should focus on accuracy and intelligibility. Finally, perhaps accreditation and certification for such training programmes can be provided to further encourage EMI lecturers to engage in continuing professional development.”*

Third, we expanded the concluding section's limitation as suggested. The added limitations include:

*“Nevertheless, the current study was limited to two private higher institutions in Malaysia and the employment of a self-reported questionnaire, which potentially produced social desirability response bias.”*

**Additional comment 1**: Introduction, p.7, 1^st^ sentence of paragraph 1 – Whom do the authors mean when they stated that EMI is “gaining traction, particularly among relevant scholars.” Who are the relevant scholars?

**Response**: Thank you for your comment, reviewer. The introduction section's first sentence was changed to reflect more clarity and the ambiguous verb "gaining traction" was eliminated:

*“The use of English as the medium of instruction (EMI) in higher education institutions is a growing phenomenon on a global scale (Dearden, 2014; Fenton-Smith, Humphreys & Walkinshaw, 2017).”*

**Additional comment 2**: Introduction, p.9, in the last full paragraph before the Literature Review section, is “academic qualification” interchangeable with “educational qualification”? Also, please clarify what it means by “diversified EMI classrooms” as this phrasing and similar such phrasing were used in the manuscript, for example, diversification of student nationalities, learner pre-knowledge level, learning styles, etc?

**Response**: Thank you very much for your suggestions to make this manuscript better. First, the term academic qualification is used consistently instead of interchangeably with educational qualification as the study refers to certifications awarded upon successful completion of a course of study in higher education (e.g., Master’s degree, Doctoral Degree).

Second, clarification on what it means by “diversified EMI classrooms” is made as suggested in the introduction section, last paragraph before the Literature Review section:

*“Subsequently, empirical findings are presented and discussed to emphasise lecturers’ important role in further enhancing intercultural communicative competence to manage the increasingly diversified EMI classrooms filled with students of various nationalities, attitudes, linguistic backgrounds, levels of prior knowledge, and learning styles.”*

**Additional comment 3**: While the authors provided a brief literature review of EMI from the perspective of lecturer training, skills, and acceptance, there was no definitions of what EMI training requirements, competencies, and knowledge base entailed. Are the competencies for gaining the skills and knowledge of EMI broad to where the skillset is transferable across all disciplines, is the approach of EMI specific for broad categories of disciplines (e.g., sciences vs liberal arts)? It would be useful to briefly note which disciplines use EMI more frequently.

**Response**: Thank you so much, reviewer. We value your input on how to make our manuscript better. Accordingly, we added a section called “Defining EMI” where we provide the variety of EMI definitions that have been found in the literature. We also next make a brief note on which disciplines use EMI more frequently, as suggested. The added section contains:

## *“****Defining EMI***

*The term EMI is unclear, even among researchers. EMI can be applied broadly, embracing any teaching strategies incorporating content in a second language. Table 1 demonstrates the variety of EMI definitions used in the scientific literature.*

***Table 1. Variety of EMI Definitions in the Literature.***

| *Year* | *EMI definitions* |
| --- | --- |
| *2011* | *“The central focus is on students’ content mastery and no language aims are specified” (Unterberger & Wilhelmer, p. 96)* |
| *2012* | *“focuses on content learning only” (Smit & Dafouz, p. 4)* |
| *2014* | *“English-taught degree programs . . . predominately aim at the acquisition of subject knowledge” (Unterberger, p. 37)* |
| *2014* | *“the use of English to teach academic subjects in countries or jurisdictions where the first language (L1) of the majority of the population is not English” (Dearden, p. 4)* |
| *2016* | *“an umbrella term for academic subjects taught through English, one making no direct reference to the aim of improving students’ English” (Dearden & Macaro, p.*  *456)* |

*According to definitions of EMI found in the literature (see Table 1), its emphasis on subject-content mastery sets it apart. Each of the interpretations shown in Table 1 highlights the value of academic content and the lack of overt language acquisition goals in EMI courses. Nonetheless, this does not imply that EMI courses cannot be designed to help improve students' English skills. The many definitions of EMI emphasise that content mastery is just one of EMI's primary goals—not its sole one.	Given the complexity of the EMI phenomenon and the range of contexts in which EMI is being introduced and developed, the concept itself is challenging to define. As a result, this study adopts the definition of EMI given below, aware that some of its elements may be contested:*

*The use of English language to teach academic subjects (other than English itself) in countries or jurisdictions where the first language of the majority of the population is not English. (Dearden, 2015, p.4)*

*The idea of “academic subjects (other than English itself)” is one particular aspect of the concept on which this study focuses. As such, it is helpful to review any prior study that identified the disciplines that use EMI the most. According to recent research by Macaro et al. (2018) that looked at a data set of 83 EMI studies, they found that engineering (n=23) and business administration and management (n=21) were the two disciplines that used EMI most frequently. However, it should be noted that some EMI studies gave broad categories of disciplines such as the sciences, social sciences, and humanities. This means that EMI usage in the two fields described above may not be entirely reflective of EMI in practice.”*

**Additional comment 4**: Methodology, p.12 – inconsistency between abstract and description in methodology: abstract stated that a survey questionnaire was administered to 227 lecturers, while Methodology section implied distribution of ”250 questionnaires in person”; rather than stating in abstract that the questionnaire was completed by 227 lecturers (out of 250 invited participants). The survey completion was noted to be voluntary and anonymous. How was anonymity preserved in receiving completed surveys when the surveys were distributed in person?

**Response**: Thank you, reviewer. We appreciate your thoughtful comment. First, the discrepancy between the methodology's abstract and the description of the number of surveys completed is clarified. Accordingly, we made it clear in the abstract that the questionnaire was completed by 227 lecturers (out of 250 invited participants). It is consistent with how we wrote for the methodology section. We concur with the reviewer that maintaining consistency is crucial.

Second, after careful consideration of the possibility of anonymity in receiving completed surveys when the surveys were distributed in person, the author realises that chances of identifying one or few participants are possible. This implies that no study carried out in person or over the phone can be regarded as anonymous. Hence, we would like to thank the reviewer for this question. Accordingly, we revise the latter part of the final sentence (underlined) in the research methods section for clarity:

*“The participation was voluntary and the participants’ data or responses were assured not to fall into the hands of a third party to ensure response confidentiality.”*

**Additional comment 5**: Table 1 noting the participants’ characteristics is not clear. What does the “Frequency” column reference – the percent of participants in the 4 disciplines noted? Recommend using a more descriptive title representing the data for the table. Recommend proofreading for consistency in verb tense used throughout (some were present tense while others were past tense) as well as run-on sentences (e.g., Results, p. 15, last sentence before Table 3 and last sentence on p.19 before Table 8).

**Response**: We appreciate the reviewer's feedback and recommendations. We agreed with the reviewer's observation and advice. First, the table's title and content have been altered to better represent the precise demographic data of participants:

***Table 2. Demographic Data of Participants.***

| *Category* | *Options* | *No. of participants* | *Percentage (%)* |
| --- | --- | --- | --- |
| *Discipline* | *Education* | *66* | *29.07* |
|  | *Linguistics* | *55* | *24.23* |
|  | *Philosophy* | *49* | *21.59* |
|  | *Psychology* | *57* | *25.11* |
| *Gender* | *Male* | *68* | *29.96* |
|  | *Female* | *159* | *70.04* |
| *Age* | *25-29* | *41* | *18.06* |
|  | *30-34* | *54* | *23.79* |
|  | *35-39* | *59* | *25.99* |
|  | *Above 40* | *73* | *32.16* |
| *Academic Qualifications* | *Master’s degree* | *133* | *58.59* |
|  | *Doctoral degree* | *94* | *41.41* |
| *Years of Teaching Experience* | *< 5 years* | *41* | *18.06* |
|  | *6-10 years* | *54* | *23.79* |
|  | *11-15 years* | *73* | *32.16* |
|  | *Above 15 years* | *59* | *25.99* |
| *Participated in EMI Training* | *Yes* | *68* | *29.96* |
|  | *No* | *159* | *70.04* |

Second, the incomplete last sentence in the results section is amended as suggested:

*“Summarily, the findings postulated that although lecturers possessed a relatively high understanding level of broader EMI aspects in higher education, a deeper knowledge of specific aspects, such as assessments, modifications to learning in a specific context, and identifications of the types of students’ EMI needs, is required as it plays a significant role in the teaching and assessment processes.”*

Finally, the author agrees with the reviewers' recommendation to double-check verb tenses. The manuscript has been proofread.

**Additional comment 6**: While much of the results were reflective of other studies, the postulate “that lecturers without adequate EMI training and preparation would be less…responsive to students’ different learning environments in content knowledge and the English language…” is unclear. How does knowledge of EMI contribute to a lecturers’ responsiveness to students regardless of learning environment, or is this in relation to a lecturer’s capability to respond in the English language?

**Response**: Thank you so much for your comment to improve our manuscript, reviewer. Accordingly, we tried to show interpreting reasons on how knowledge of EMI contributes to a lecturers’ responsiveness to students’ different learning environments in the discussion section (third paragraph, final sentence):

*“This further suggests that having adequate EMI knowledge helps lecturers be sensitive in terms of their capacity to appropriately respond to the particular interaction and scenarios in English.”*

**Additional comment 7**: It would be interesting to learn of a follow-up from this study after an intervention to provide internal faculty/professional development on EMI and resource support using this same survey - how would the attitudes have changed based on participant characteristics? (For both private and public institutions)

**Response**: Thank you very much, reviewer, for your interest and suggestion. We gladly accept your suggestion and accordingly, we included it in the discussion section (final paragraph):

*“Accordingly, a follow-up intervention of offering internal faculty/professional development on EMI and resource assistance utilising this same survey for private and public institutions may be appropriate.”*
